# Using the Analytic Hierarchy Process (AHP) to understand the most important factors to design and evaluate a telehealth system for Parkinson's disease

**DOI:** 10.1186/1472-6947-15-S3-S7

**Published:** 2015-09-04

**Authors:** Jorge Cancela, Giuseppe Fico, Maria T Arredondo Waldmeyer

**Affiliations:** 1 Life Supporting Technologies, Universidad Politecnica de Madrid, ETSI Telecomunicación, Ciudad Universitaria, Madrid, Spain

## Abstract

**Background:**

The assessment of a new health technology is a multidisciplinary and multidimensional process, which requires a complex analysis and the convergence of different stakeholders into a common decision. This task is even more delicate when the assessment is carried out in early stage of development processes, when the maturity of the technology prevents conducting a large scale trials to evaluate the cost effectiveness through classic health economics methods. This lack of information may limit the future development and deployment in the clinical practice. This work aims to 1) identify the most relevant user needs of a new medical technology for managing and monitoring Parkinson's Disease (PD) patients and to 2) use these user needs for a preliminary assessment of a specific system called PERFORM, as a case study.

**Methods:**

Analytic Hierarchy Process (AHP) was used to design a hierarchy of 17 needs, grouped into 5 categories. A total of 16 experts, 6 of them with a clinical background and the remaining 10 with a technical background, were asked to rank these needs and categories.

**Results:**

*On/Off fluctuations detection, Increase wearability acceptance*, and *Increase self-management support *have been identified as the most relevant user needs. No significant differences were found between the clinician and technical groups. These results have been used to evaluate the PERFORM system and to identify future areas of improvement.

**Conclusions:**

First of all, the AHP contributed to the elaboration of a unified hierarchy, integrating the needs of a variety of stakeholders, promoting the discussion and the agreement into a common framework of evaluation. Moreover, the AHP effectively supported the user need elicitation as well as the assignment of different weights and priorities to each need and, consequently, it helped to define a framework for the assessment of telehealth systems for PD management and monitoring. This framework can be used to support the decision-making process for the adoption of new technologies in PD.

## Background

Parkinson's disease (PD) is a neurodegenerative disorder affecting more than 1% of people older than 60 years (and with an increased prevalence in older subjects). As other chronic diseases, PD is a prolonged condition that does not improve with time and cannot be cured completely. Progression of the disease is strongly correlated with higher costs - for both patients and healthcare institutions - and substantial savings can be achieved by slowing down this progression [1].

Telemedicine could contribute to optimize the use of health services in PD management by delivering care services in daily living environments and strengthening the connection between patients and care providers. This would result in improved level of care measured through target indicators like number and quality of visits, access to emergency departments and ambulance services, number of hospitalizations, hospital readmissions, and length of hospital stay, number of referrals, duration of consultations, number of laboratory tests, and avoided transfers/evacuations [2,3]. Yet, there is a widespread perception that telemedicine applications are not as widely used as expected. The limited availability of information on large-scale performance and economic impact might account for some of these perceptions [4,5]. This is particularly relevant in the case of PD, where no large scale pilots have been conducted in order to address these issues, as most validations are limited in number of subjects and are focused on the assessment of the technical feasibility and clinical outcomes [6].

To estimate the economic benefits of telemedicine programs, clinical and social outcomes must be translated into monetary values using reliable conversion factors, but before that, it should be verified that the technology properly satisfies user needs. This step is problematic and complex in healthcare, which by nature is a multidisciplinary area involving multidimensional data sources and stakeholders. Designers and developers of a new technology have, in general terms, a more advanced knowledge in the technological field than the final users. Therefore, it is fundamental to elicit end-users perspectives during the design process, and take them into account for the assessment of the technology once it is developed and tested.

Additionally, different aspects and factors may affect the user needs and the problem definition: medical goals are often driven by contingencies, they change according to the scale of the problem, and therefore it is not easy to identify a gold standard method. Although many authors suggest to assess the effects of technology-based healthcare interventions by grouping them in several dimensions (economical, technical, ethical, etc.), the majority of studies are focused mainly on two of them, the clinical and the economical ones [6].

It is widely accepted that medical devices, or in general health technologies can be used only if they cover the needs of end-users [7]; achieving this goal can be complex and challenging: identical requirements (e.g. safety, efficacy) can be evaluated differently for the same technology and technique, depending on the clinical problem, the medical and organizational skills and procedures of a practice team, on patient's attitudes and health status [8,9]. Even within apparently homogeneous user groups, individuals may have received different training and working guidelines. Opinions from different stakeholders must be collected, considered and balanced according to the differences and conflicts that may arise. This issue is relevant for professionals involved in decision making processes related with the design, production, evaluation and purchase of medical devices [9]. As a matter of fact, the healthcare industry has been increasingly adopting and making use of user needs elicitation methods and seeking for quantified and objective information to define plans and strategies for improving their service/product portfolios and reduce their market risks [10].

Nevertheless, the lack of time and/or resources, as well as the lack of knowledge about appropriate methods for data collection and analysis could compromise the elicitation of requirements [11,12], resulting in information base that could be fragmented, partial, inconsistent, not meaningful and as such, not enough to answer the desired questions [9,13].

This research work aims at identifying and ranking the key user needs that a telehealth system for the management of Parkinson's disease should cover, in order to be useful and effective. Additionally to the identification, a quantitative evaluation of the importance of each need compared to the others is provided. The Analytic Hierarchy Process (AHP) was chosen to perform this work. This methodology has been already used in healthcare, with different goals and objectives, such as user needs elicitation [9,14,15], medical decision-making [16], budget allocation [17] and medical device purchasing [18]. A group of experts was involved to identify and prioritize these needs. Two types of expertise have been required as inclusion criteria for the expert selection. On the one hand, participants with a clinical background, and with experience and knowledge of the eHealth field; on the other hand, participants with a technical background as well as experience in the design and development of telehealth and/or eHealth systems. In both cases, the selection of the participants required at least a basic knowledge of the Parkinson's disease symptoms and progression. The analysis of the results allowed to identify, rank and quantify the views from both technical and clinical experts, and finally, to understand in which cases their opinion is similar and in which cases there are discrepancies in their points of view.

As a case study, the results this process have been used for the assessment a telehealth system for Parkinson's disease management called PERFORM [19].

### Parkinson's disease

PD is a disorder largely caused by the loss of dopaminergic innervation of the basal ganglia, resulting in motor disturbances such as slowed movement (*bradykinesia*), small amplitude movements (*hypokinesia*), resting tremor, postural instability and rigidity.

These problems often restrict functional independence and are a major cause of morbidity and mortality among these patients [20-23]. PD is typically characterized by severe, unpredictable and abrupt changes in the patient motor performance whereby OFF periods, characterized by the temporary loss of drugs effectiveness, alternate, sometimes within minutes, with ON periods, during which the medication effectively attenuate motion symptoms.

### PERFORM system

PERFORM system is focused on achieving a better medication adjustment in PD through a better and more objective monitoring of the patients' symptoms (specially through a better identification of the On-Off fluctuations), reducing costs by utilizing Information and Communication Technologies (ICT), reducing the number of unneeded transportations and optimizing the waiting and consultation time, transferring health knowledge from the practitioner to the patient, increasing self-management capability and medication adherence. The system includes a set of wearable sensors for the continuous recording of the motion signals and a set of software algorithms for the signal processing. The wearable system is composed of a set of four wearable tri-axial accelerometers placed at each patient limb (wrists and ankles) used to seamlessly record acceleration in the three spatial directions; a waist sensor, composed of an accelerometer and a gyroscope; and a data logger used to receive and store all the data in a SD card. Data transmission from the sensors to the data logger was done using Zigbee protocol and the sampling rate was 62.5 Hz. All accelerometers transmit data simultaneously and without retransmission of lost packets, in order to save battery. The system is characterized with a global data loss of 1.24 ± 0.58% [24].

A specific software system, the Local Base Unit (LBU), was built and installed at patients' homes. It was responsible for the automatic detection and quantification of the patient symptoms - based on the data download from the data logger - and the recording of other useful information for the evaluation of the patient status. For each symptom, a dedicated algorithm processes the relevant signals, detects the symptom episode and quantifies it into a severity scale from 0 to 4, according to the Unified Parkinson's Disease Rating Scale (UPDRS). The Graphical User Interface (GUI) used in the LBU was specifically designed for PD patients, allowing them to enter information, like medication intakes (type, dose and time), meals (type of food, amount, time) and PDQ-39, a standard questionnaire for the evaluation of physical, emotional and psychosocial aspects of Quality of Life (QoL) in PD patients [25,26]. The GUI was tested in different phases and redesigned according to the users' feedback [27].

Regarding the technical performance of the system, it shows an accuracy of up to 93.73% for the classification of levodopa induced dyskinesias (LID) severity [28], an 86% for the classification of bradykinesia severity [29] and 87% for tremor severity [30]. Also, a specific module was developed for the assessment of gait [31] and for the detection of On-Off fluctuations [32] (showing an average accuracy of 88.2% in classifying the time that the patient spends in the different phases).

## Methods

### Ethical considerations

This was an interview study with experts and without patient involvement. A participant information sheet was presented informing about the purpose of study and a detailed description of the tasks that they must perform. Participants gave informed consent to participate in the study. The data collected was associated to the participant profile and background in order to analyse the results properly. The name or any other personal detail has not be disclosed at any time.

### AHP

AHP is a method for decision-making, aiming at solving complex problems. This method allows quantifying opinions and transforming them into a coherent decision model. A hierarchy of elements, grouped into categories, is defined through this method. Elements and categories are then ranked, via questionnaires, through pair-wise comparisons. Moreover, it is possible to assess the coherence of respondent judgments and ask the experts to refine incoherent answers. Once questionnaires are finalized, it is possible to extract the relative importance of each need per category (local weights, LW), the relative importance of each category (category weights, CW), and the importance of each need compared to all the others (Global weights, GW) [33,34].

### Hierarchy definition

In order to identify the elements and categories of the hierarchy, a literature review was performed; then a focus group with experts was done to organize these factors in nodes and leafs. This focus group involved 2 clinicians and 5 biomedical engineers with experience in the design, assessment and management of medical devices, of which 2 are co-authors of this paper (JC, GF). JC acted as the facilitator and designed the first version of the hierarchy, based on his own experience, and the work of Dávalos et al. [2], who proposed a wide range of indicators that can be used to analyse the outcome of telemedicine systems, and how to convert them into monetary units. Then, the hierarchy was distributed and reviewed by the other experts. Finally, it was updated according to the feedback received from all of them. In the first iteration, 29 needs were identified and organized into five meaningful categories. Then, common needs were merged, in order to avoid overlapping and thus optimize the total number of needs. This process has been presented in [35]. The final tree is described in Table 1; each node represents a category, each leaf represents a need. It is composed of 5 categories and 17 needs.

**Table 1:** AHP hierarchy.

Nodes (Categories)	Elements (User needs)
Performance	Motor symptoms assessment
	
	ON/OFF fluctuations detection
	
	Cognitive & behavioral assessment
	
	Data mining & disease modelling

User experience	↑ wearability acceptance
	
	User-friendly interfaces
	
	Seamlessly integration

Clinical practice	↑ patient-clinician bond
	
	↑ patient & carers knowledge
	
	↑ self-management support
	
	↑ assist care givers

Economic	↓ visits and stays in hospital
	↑ patient Quality of Life
	
	Faster and more reliable diagnosis

Technical issues	Scalability and interoperability
	
	Security and privacy
	
	↓ maintenance and support cost

Hierarchy used for this AHP study including the categories and user needs.

### Participants

Table 2 shows the profile of the different responders involved in this study.

**Table 2:** Participants profile.

Code	Profile	Sex	Years of working experience	Years of experience with PD	Years of experience with eHealth
1	Technical	Female	26	5	26

2	Technical	Male	20	11	20

3	Technical	Male	8	7	8

4	Technical	Male	12	6	12

5	Technical	Male	7	5	6

6	Technical	Male	7	5	6

7	Technical	Male	10	1	8

8	Technical	Male	10	2	8

9	Technical	Male	5	4	5

10	Technical	Male	7	5	6

11	Clinical	Female	8	4	5

12	Clinical	Female	12	10	4

13	Clinical	Male	19	11	5

14	Clinical	Male	25	25	15

15	Clinical	Female	26	12	0

16	Clinical	Female	4	4	2

Profile of the participants in the AHP study

### Questionnaires

Once the tree was finalized, questionnaires were prepared to compare the relative importance of each need with all of the other needs within the same category. For each pair of needs, the following question was asked: "In a technological solution for Parkinson's disease monitoring, assessment and/or management, from your point of view and according to your experience, which is the most important element and how much important it is with respect to the other?". Responders answered by choosing which one of the options was more important, by selecting an integer numerical value from 1 (both options are equally important) to 9 (the selected element is much more important).

The application used for creating the hierarchy and collecting answers is the BPMSG AHP Online System (Available online: http://bpmsg.com/). This system automatically calculates the Consistency Rate (CR) and shows some options to improve the consistency when the value is higher than 10%.

### Grouping experts judgements

**Figure 1:**
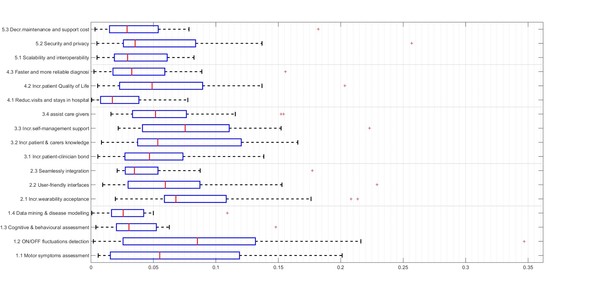
**Boxplot with the result of all the responders**. Central mark is the median, the edges of the box are the 25th and 75th percentiles, the whiskers extend to the most extreme data points not considered outliers, and outliers are plotted individually.

**Figure 2:**
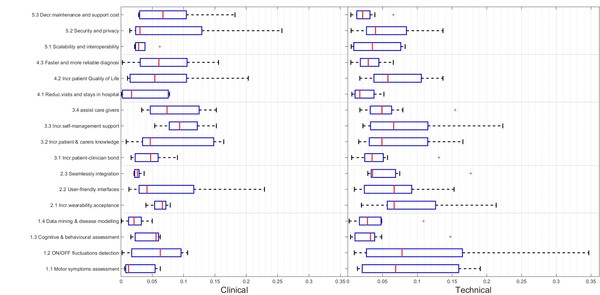
**Boxplot with the result of the responders grouped by technicians and clinicians profiles**. Central mark is the median, the edges of the box are the 25th and 75th percentiles, the whiskers extend to the most extreme data points not considered outliers, and outliers are plotted individually.

One of the aims of this work was to explore the differences between the two groups of experts. Different actions were done to study the different perspectives of both groups. The first one was to graphically represent the GW of each user needs using a boxplot chart with data from all the participants (Figure 1) and another one was to aggregate the GW with the technical and clinical participants (Figure 2). Boxplots plot the median values (central marks), the 25th and the 75th percentiles (the blue range) and the outliers (crosses). Then, on the different results tables (Table 3 and Table 4) the median values of the GW, LW and CW for each group are included in a numerical way.

**Table 3:** Local and global weights of needs (CR<0.1), median of the Local and global weights for the clinical and technical groups and t-test between the two groups.

	Group results GW (LW)	Median Technical GW (LW)	Median Clinical GW (LW)	GW t-test (LW t-test)
1 Performance				

1.1 Motor symptoms assessment	0.064 (0.292)	0.064 (0.304)	0.032 (0.231)	0.522 (0.486)

1.2 ON/OFF fluctuations detection	**0.085 **(**0.388**)	**0.099** (**0.392**)	**0.078** (0.288)	0.269 (0.24)

1.3 Cognitive & behavioral assessment	0.042 (0.197)	0.030 (0.127)	0.041 (**0.305**)	0.844 (**0.032**)

1.4 Data mining & disease modelling	0.028 (0.129)	0.027 (0.090)	0.024 (0.120)	0.448 (0.664)

2. User experience				

2.1 ↑wearability acceptance	**0.102 **(**0.457**)	**0.077** (**0.388**)	**0.068** (**0.461**)	0.782 (0.494)

2.2 User-friendly interfaces	0.068 (0.305)	**0.077** (0.281)	0.047 (0.310)	0.849 (0.408)

2.3 Seamlessly integration	0.053 (0.237)	0.038 (0.322)	0.026 (0.169)	0.099 (0.107)

3 Clinical practice				

3.1↑patient-clinician bond	0.054 (0.181)	0.041 (0.203)	0.048 (0.139)	0.695 (0.736)

3.2 ↑patient & carers knowledge	**0.080** (0.270)	0.054 (0.25)	0.063 (0.245)	0.761 (0.593)

3.3 ↑self-management support	**0.093** (**0.314**)	0.058 **(0.290)**	**0.089** **(0.284)**	0.880 (0.849)

3.4 assist care givers	0.070 (0.235)	0.051 (0.201)	0.062 (0.259)	0.482 (0.814)

4 Economic				

4.1 ↓visits and stays in hospital	0.022 (0.175)	0.015 (0.169)	0.027 (0.162)	0.313 (0.588)

4.2 ↑patient Quality of Life	0.063 (**0.511**)	0.051 **(0.468)**	0.035 **(0.376)**	0.826 (0.197)

4.3 Faster and more reliable diagnosis	0.039 (0.313)	0.029 (0.299)	0.050 (0.323)	0.189 (0.428)

5 Technical issues				

5.1 Scalability and interoperability	0.040 (0.293)	0.039 (0.310)	0.026 (0.281)	0.472 (0.615)

5.2 Security and privacy	0.059 (**0.430**)	0.041 **(0.464)**	0.029 **(0.396)**	0.563 (0.600)

5.3 ↓maintenance and support cost	0.038 (0.277)	0.024 (0.177)	0.049 (0.367)	0.092 (0.341)

**Table 4:** Categorical weights, median of the weights for the clinical and technical groups and t-test between the two groups.

	CW	Technical	Clinical	t-test
1 Performance	0.219	0.207	0.200	0.375

2 User experience	0.222	0.216	0.159	0.560

3 Clinical practice	**0.297**	**0.233**	**0.278**	0.573

4 Economic	0.124	0.110	0.104	0.520

5 Technical issues	0.137	0.103	0.140	0.337

### Correlations among responders' preferences and group consensus

In order to analyse the consistency of the responses between the different participants, two different measures have been used. The first one is the Spearman rank correlation (ρ or RHO), commonly used for AHP-based studies [9,36,37], to show how much two sets of elements are ranked in the same order [38]. The p-value was used (p < 0.05) to identify which values should be considered as significant.

Furthermore, the BPMSG AHP Online System automatically provides a measure of consensus group (both for the general hierarchy as well as for the responses of every individual category), defined as *a diversity index for the distribution of priorities among criteria *[39]. The diversity index is then used to build a relative index of homogeneity S, which can be used as a consensus indicator; it is zero, when the priorities are completely distinct and unity when the priorities of all are identical.

## Results

**Figure 3:**
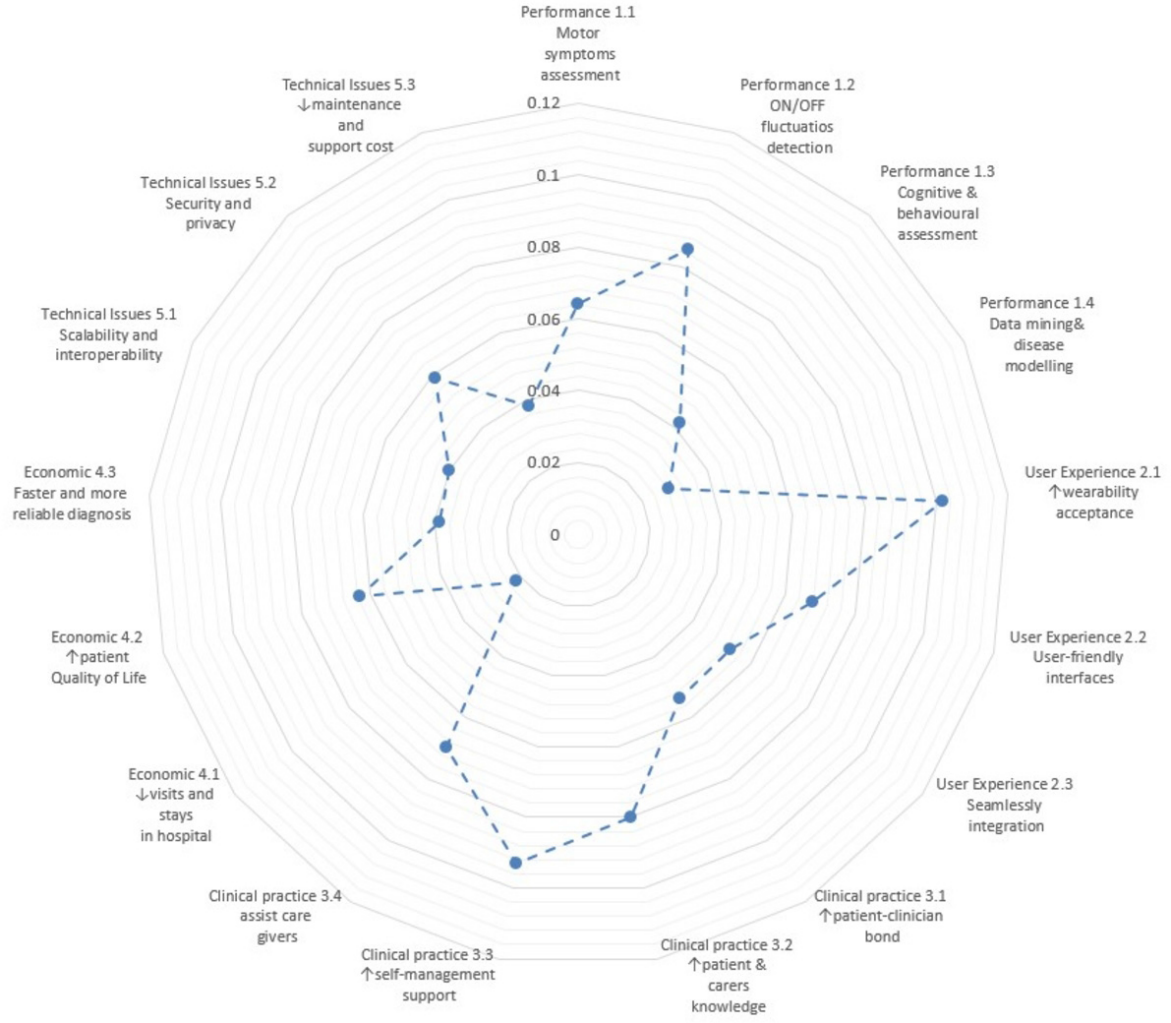
**Group results of the user needs**. This chart shows the group results of the GW for each user need including the response of all the participants.

Figure 3 shows the global picture of the AHP results and Table 3 summarizes the results of the AHP questionnaire. The second column shows for each element of the hierarchy the Global Weights (GW) and the Local Weights (LW) resulted from the AHP analysis. These results are based on the AHP method and take into account the responses from all the participants. The third and fourth columns show the median of the GW and LW values, grouped according to the profile of the responders (clinical and technical). The last column show the t-test comparing the responses of the two groups of responders. The information of the median values is also provided in the Figure 1, where the boxplots show, for each element of the hierarchy, the median value (central mark), the 25^th ^and the 75^th ^percentiles.

Table 4 contains the Categorical Weights (CW) obtained from the responses of all the participants (second column); the third and fourth columns show the median values obtained for the technical and clinical groups. The last column shows the t-test between the CW between clinical and technical groups.

Table 5 shows the group consensus within each category, within categories and integrated group consensus based on the GW values.

**Table 5:** Group consensus.

	Group consensus
1 Performance elements	0.720

2 User experience elements	**0.867**

3 Clinical practice elements	0.773

4 Economic elements	0.762

5 Technical issues elements	0.784

Between categories elements	0.709

AHP Group consensus (rel. homogeneity )	0.736 (0.734)

## Discussion

This work has two main goals: firstly, to identify the elements that a telehealth system for PD management should fulfil in order to be effective and, secondly, to understand if clinicians and technicians share a common opinion when evaluating these systems.

According to the results of the responders, the three most important user needs are *Increase the wearability acceptance *(0.102), *Increase the self-management support *(0.093) and *On/Off fluctuations detection *(0.085), based on the values from the Global Weights (GW). In a second level of importance we found *Increase patient & carers knowledge *(0.080), *Assist care givers *(0.070), *User-friendly interfaces *(0.068), *Motor symptoms assessment *(0.064) and *Increase patient Quality of Life *(0.063).

The analysis of the Categorical Weights (CW) provides information about the importance of the different categories. The *Clinical Practice *category has been highlighted as the most relevant category by all the responders (0.297), followed by the *Performance *and *User experience *categories which scored very similarly (0.219 and 0.222). Finally, the *Economic *and *Technical Issues *categories were the less relevant according to these results (0.124 and 0.137). As regards the differences between the clinical and the technical groups, in both cases the Clinical practice category has the highest relevance. *Performance *and the *User experience *categories as the following categories, while *Economic *and *Technical issues *as the less relevant ones. Nevertheless, there are some differences: for the technical group the *User experience *is the second most important category and the *Technical issues *the less important; for the clinical group it is the opposite: *Performance *is the second most important, while the *Economic *category the less one. However, no significant difference between the responses of the clinical and technical groups has been found. Regarding the GW and LW of all the elements (Table 3), a similar scenario has been found. In this case, the only statistically significant difference is found in the LW of the *Cognitive & behavioural modelling *element. The clinical group has rated this issue significantly higher than the technical group. In all the other cases, the differences are not significant. Besides, in the clinical group the three top GW of the clinical group match the aggregated top three GW; in the case of the technical group, *On/Off fluctuations detection, Increase wearability acceptance*, and *User-friendly interfaces *have been considered as the most relevant elements. It is also important to highlight that some of those requirements such as wearability, usability have a huge impact, not only on the user experience but also on the effectiveness and safety of health technologies.

As regards the relevance of the different elements given a specific category: in the *Performance *category *On/Off fluctuations detection *(0.388) is the most important element, followed by the *Motor symptoms assessment *(0.292), *Cognitive & behavioural *assessment (0.197) and *Data mining & disease modelling *(0.129). Regarding the *User experience *category, *Increasing the wearability acceptance *(0.457) is considered the most important need, followed by *User-friendly interfaces *(0.305) and *Seamlessly integration *(0.237). According to these results, when working with wearable devices, the User Experience (UX) is strongly affected by the experience of the user interacting with the physical devices. This effect is due to not only to the physical features of the devices (i.e. size, weight, comfort) but also to a social component implicit in wearing a medical device (i.e. what other people could think when I wear this device) [40]. This issue may also be linked with the privacy of the patient.

In the third category, *Clinical practice*, the need *Increase self-management support *(0.314) obtained the highest LW, followed by *Increase patient & carers knowledge *(0.270) and *Assist care givers *(0.235). Finally, *Increase patient-clinician bond *(0.181) scored the lowest weight. These results reflect a clear demand in providing both patients and carers with tools that improve the management of the disease and increase the effectiveness of healthcare interventions.

As regards the fourth category, *Economic *aspects, *Increase patient Quality of Life *(0.511) is considered as the most important element; the second one is *Faster and more reliable diagnosis *(0.313), while *Decreasing visits and stays in the hospital *(0.175) is considered as less important. This last result was expected since most of the PD patients do not suffer acute episodes requiring long stays in the hospital. Instead, the main economic benefit could be achieved by improving the QoL. Actually, this could also be expected since PD has an enormous impact on the QoL by limiting their mobility and independence. Additionally, improving in diagnosis could also generate an economical benefit due to a most effective use of the resources.

Finally, within the last category, *Technical issues*, the *Security and privacy *(0.430) aspects are ranked as the most relevant user needs, followed by *Scalability and interoperability *(0.293) and *Decrease maintenance and support cost *(0.277). As expected in a healthcare scenario, security and privacy issues are the most relevant needs, while other Information Technology (IT) issues are considered as secondary.

Usually, clinical effectiveness and safety are the primary concerns in medicine [8], in this case, the impact of a telehealth system for disease management and monitoring in the safety of the patient is very limited and this fact is reflected in the opinion of the experts that did not include these issues among the most relevant ones.

Additionally to the t-test performed between the median values for the CW, GW and LW calculated for the clinical and technical groups, the Spearman correlation (ρ) and the associated p-value (p) were also calculated taking into consideration the median values of the GW for the clinical and technical groups. This measure is intended to quantify the similarity between the responses of both groups. The result is ρ = 0.590 (p = 0.012): we can conclude that there is a significant correlation between both groups. This fact is reinforced by the measures of the group consensus on Table 5. These values show a high degree of consensus between all the participants, especially within the elements of the *User experience *category (0.867).

### Evaluation of the PERFORM system

In additional file 1, Table S1, a description of the user needs identified in the AHP and to which extent the PERFOM fulfils them is given. This way, it is possible to evaluate the PERFORM system by understanding if the system complies with each needs and estimating its importance, weighted according to the GW of each need.

As regards the most relevant elements, *On/Off fluctuations detection, Increase wearability acceptance, Increase patient & carers knowledge *and *Increase self-management support*, it is possible to appreciate that the PERFORM system fulfilled reasonably the *On/Off fluctuations detection *and *Increase wearability acceptance*. It partially complies with the *Increase self-management support*. Nevertheless, it failed in reaching the *Increase patient & carers knowledge *element: even though the system provides enough information about patients' symptoms and their evolution, it does not give support in understanding their own status. According to the experts, it is not enough to monitor and assess the patient seamlessly but it is also crucial to advise and guide him/her, by customizing and personalizing guidelines according to the current status that the system allows to assess. Another important issue where PERFORM fails and that it was highlighted as important, is to *assist care givers*. According to the experts consulted in the study, the role of caregivers is remarkably important - this stakeholder is also included in the *Increase patient & carers knowledge - *and was not considered in the current version of the system.

Moreover, PERFORM did not incorporate the monitoring of no-motor systems, being especially relevant the *Cognitive & behavioral assessment one*. The incorporation of these user needs and the jointly assessment of motor and cognitive components would significantly enrich and benefit the modeling of the disease according to the experts' opinion.

## Conclusions

First of all, AHP contributed to the elaboration of a common hierarchy, integrating user needs from different stakeholders and promoting the discussion and the agreement into a common framework. Moreover, AHP effectively supported the user need elicitation as well as the assignment of different weights and priorities to each need and consequently, it helped in the elaboration of common framework of assessment of telehealth systems for PD management and monitoring. It has been used to clearly identify the key user needs that a telehealth system for the remote monitoring and management of PD should fulfil, specifically, the *On/Off fluctuations detection, Increase wearability acceptance, Increase self-management support *and *Increase self-management support *have been identified as the most relevant by a group of clinicians and engineers with experience in the field.

Thus, this work offers a validated framework of evaluation for researchers and developers working in the field of telehealth for PD management and assessment a useful tool for the identification of improvement areas. In the case of the PERFORM system three areas of future improvement were identified: 1) the involvement of the carers in the loop was strongly recommended according to the AHP results, 2) the telehealth system and the information from the monitoring and assessment should be used to educate and feedback patient and carers according to their specific needs in order to make the platform actually useful and 3) the incorporation of cognitive and behavioral analysis to the motor assessment may significantly enhance the modeling of the disease. These elements have been considered for the design of a new research project, the PD Manager project (http://www.parkinson-manager.eu/), in which the role of the caregivers, behavioural and cognitive monitoring and patient education have been included as important pillars for a new generation of mHealth services for Parkinson disease. A second conclusion is that no significant differences were found between the clinician and technical groups. Moreover, AHP proved to be a useful tool to improve the communication and promote the discussion among the different stakeholders and the different professionals involved in the design of telehealth systems. In this case a clear agreement between both groups were found and only one of the seventeen user needs show a slight discrepancy between these groups (only at a LW level). Moreover, the high levels of correlation within the answers and of group consensus confirm that this framework could be useful for future evaluation of technologies in PD and thus support the decision-making process in their adoption.

## Competing interests

JC, GF and MTA are affiliated with Universidad Politecnica de Madrid, which has been part of the PERFORM Consortium and it is currently part of the PD_manager Consortium. JC and MTA have been directly involved in the research activities of the PERFOM and PD_manager projects.

## Authors' contributions

JC and GF conceived this study. JC drafted the hierarchy and the questionnaires, analyzed the data and presented the results. JC, GF and MTA discussed the results considering the state of the art of the literature, drafted the paper and reviewed the manuscript. All authors read and approved the final manuscript.

## Supplementary Material

Additional file 1 **Table S1, additional file 1- Evaluation of PERFORM system**. Click here for file

## Declarations

Publication costs have been funded by the PD_manager project.

This article has been published as part of *BMC Medical Informatics and Decision Making *Volume 15 Supplement 3, 2015: Multidimensional, multidiscipline and shared decision making in healthcare and eHealth. The full contents of the supplement are available online at http://www.biomedcentral.com/bmcmedinformdecismak/supplements/15/S3.
