# Supplementary material for: Using the Analytic Hierarchy Process (AHP) to understand the most important factors to design and evaluate a telehealth system for Parkinson's disease
Source: BMC Med Inform Decis Mak. 2015 Sep 4;15(Suppl 3):S7. doi: 10.1186/1472-6947-15-S3-S7 (PMC4705498; doi:10.1186/1472-6947-15-S3-S7)
Supplement: Additional file 1 — Table S1, additional file 1- Evaluation of PERFORM system. [file 1472-6947-15-S3-S7-S1.docx]

Table S1

| User needs | GW | PERFORM |
| --- | --- | --- |
| 1 Performance |  |  |
| 1.1 Motor symptoms assessment | 0.064 | One of the main objectives of the PERFORM system was the automatic detection and assessment of the different PD symptoms. Specific algorithms were developed in order to assess bradykinesia, dyskinesias, freezing of gait, gait performance and tremors.  About the technical performance of the system it shows an accuracy of up to 93.73% of accuracy for the classification of Levodopa Induced Dyskinesias (LID) severity [28], an 86% for the classification of bradykinesia severity [29] and 87% for tremor severity [30]. Also, a specific module was developed for the assessment of gait [31]. |
| 1.2 ON/OFF fluctuations detection | **0.085** | The detection of the On and Off phases was a main goal for the project. A detection system was designed based on the fluctuations of the motor symptoms previously assessed. This module showed an average accuracy of 88.2% classifying the time that the patient spend on the different On and Off phases [32]. |
| 1.3 Cognitive & behavioral assessment | 0.042 | Cognitive and behavioral assessment was not included in the PERFORM system |
| 1.4 Data mining & disease modelling | 0.028 | PERFORM also included a platform for the clinicians and for the healthcare professionals where all the information coming from the continuous monitoring of the patients is used to build a tailored model of the disease and generate alerts when a remarkable event was detected [19]. This rule-based system takes not only the motor assessment but also other information provided by the PERFORM system such as food and medication intakes [41]. |
| 2. User experience |  |  |
| 2.1↑wearability acceptance | **0.102** | The wearability acceptance of the system was seriously taking into account during the project. Actually, this issue was also raised during the evolution of the project, external reviewers suggested that a specific strategy to study and address this topic would benefit enormously the project, for this reason a wearability study was carried out [42]. In this study most of the patients showed their positive compliance with the wearable devices and only some subject reported issues regarding the size and shape of the sensors [19]. A variety of methodologies (REBA, Borg and CRS scales in combination with a body map) has been used to study the comfort, biomechanical and physiological effects of the system. The acceptance of the system was eventually satisfactory and user-friendly, over the desired threshold. Moreover, useful insights and guidance to improve the system design were gathered in this phase. |
| 2.2User-friendly interfaces | 0.068 | PERFORM followed a User-Centered design approach both with clinicians and patients. Several redesigns of the Graphic User Interfaces (GUI) were done following the feedback of the users on each iteration [19]. |
| 2.3 Seamlessly integration | 0.053 | The PERFORM system was designed following seamlessly integration as one of the technical requirements. Download of data from sensors to the patient computer, processing of data and transmission of data to the hospital works seamlessly. Nevertheless switch on the monitoring sensors require some effort by the users [19]. |
| 3 Clinical practice |  |  |
| 3.1↑patient-clinician bond | 0.054 | PERFORM facilitates the continuous monitoring and the data transmission from the patient to the clinician, as well as it enables and facilitates a better knowledge of the patient by the clinician and healthcare professionals. Nevertheless, in the opposite direction it only allows the clinician to request a new appointment but does not allow them to establish a richer communication [19]. |
| 3.2↑patient & carers knowledge | **0.080** | PERFORM system was designed to perform a seamlessly monitoring and, since one the aims of the projects was the validation of such assessment methodologies, it was decided to do that without providing a specific feedback to the patients about their assessment or the evolution of the disease. Instead, all the monitoring data was sent directly to the hospital where a rule-based Decision Support System (DSS) alert the clinicians in the case of the symptoms fluctuated excessively. |
| 3.3↑self-management support | **0.093** | Using PERFORM the patient takes a more active role in the assessment and monitoring of his own disease – for example by reporting medication and food intakes. They were also trained to use the wearable sensors for the daily monitoring. In this sense, the patients take an active role in the self-monitoring of their own disease – still, it is insufficient. More feedback and a clear and tailored management guidelines may be generated and presented to the patient in order to support him on this process [19]. |
| 3.4 assist care givers | 0.070 | Care givers have not been included as potential stakeholders in the design of the PERFORM project. In this sense, most of the user needs of the care givers are not covered. |
| 4 Economic |  |  |
| 4.1↓visits and stays in hospital | 0.022 | The automatic detection of motor symptoms and On-Off fluctuations allows the adjustment of the medication and the reduction of Off periods without the necessity of a visit to the hospital. Also, the continuous monitoring of motor symptoms and the automatic generation of a report enable the remote monitoring and follow-up of the patients [19]. On the other hand, even if visits and stays may be reduced PERFORM will require a trained professional working on the tool continuously. |
| 4.2↑patient Quality of Life | 0.063 | The actually impact patient QoL it is needed to keep the patient on the On state during as much time as possible during the day. The automatic detection of motor symptoms and On-Off fluctuations allows the adjustment of the medication and the reduction of Off periods and consequently the increase of patient QoL. |
| 4.3Faster and more reliable diagnosis | 0.039 | As it was abovementioned PERFORM allow the continuous monitoring and assessment of the motor symptoms of PD patients. Therefore, it provides clinicians with an objective tool for the diagnosis of PD patients. |
| 5 Technical issues |  |  |
| 5.1 Scalability and  interoperability | 0.040 | The last prototype of PERFORM was developed based on market standards. The patient application was developed on .NET, the sensors download information to the LBU application through a USB and the clinicians’ application is on the cloud working as a web application from any browser. This need can be improved by the adoption of healthcare standards (e.g. HL7). |
| 5.2 Security and privacy | 0.059 | PERFORM was designed following security and privacy guidelines for healthcare applications. SSL was used for the transmission of data between the patients’ home and the hospital solutions. |
| 5.3↓maintenance and support cost | 0.038 | No specific actions were taking on this regards. A large pilot is required to accurately measure the technical maintenance and the support cost of the PERFORM system and to compare it with the current clinical practice. |
